# Metabolic Adaptations in Cancer and the Host Using *Drosophila* Models and Advanced Tools

**DOI:** 10.3390/cells13231977

**Published:** 2024-11-29

**Authors:** Ernesto Saez-Carrion, Mario Aguilar-Aragon, Lucia García-López, Maria Dominguez, Mary Luz Uribe

**Affiliations:** 1 Instituto de Neurociencias, Consejo Superior de Investigaciones Científicas (CSIC), Universidad Miguel Hernández (UMH), Campus de Sant Joan, 03550 Sant Joan d’Alacant, Spain; mario.aguilar@umh.es (M.A.-A.); lucia.garcia@universidadeuropea.es (L.G.-L.); m.dominguez@umh.es (M.D.); 2 Faculty of Health Sciences, Universidad Europea de Valencia, 03016 Alicante, Spain

**Keywords:** cancer, metabolism, diet, inter-organ communication, microenvironment, systemic, metastasis, omics, technologies, anti-cancer drugs

## Abstract

Cancer is a multifactorial process involving genetic, epigenetic, physiological, and metabolic changes. The ability of tumours to regulate new reactive pathways is essential for their survival. A key aspect of this involves the decision-making process of cancer cells as they balance the exploitation of surrounding and distant tissues for their own benefit while avoiding the rapid destruction of the host. Nutrition plays a central role in these processes but is inherently limited. Understanding how tumour cells interact with non-tumoural tissues to acquire nutrients is crucial. In this review, we emphasise the utility of *Drosophila melanogaster* as a model organism for dissecting the complex oncogenic networks underlying these interactions. By studying various levels—from individual tumour cells to systemic markers—we can gain new insights into how cancer adapts and thrives. Moreover, developing innovative technologies, such as high-throughput methods and metabolic interventions, enhances our ability to explore how tumours adapt to different conditions. These technological advances allow us to explore tumour adaptations and open new opportunities for potential therapeutic strategies.

## 1. Introduction

Cancer, a relentless survival strategy of a single cell, is a pressing global health issue. By 2019, it claimed the top spot as the leading cause of death in 57 countries and the second spot in 55 others in individuals under 70 years old [1]. The intricate oncogenic network, encompassing inner cellular changes and their potential impact, has been meticulously documented and updated over the decades [2,3,4].

Among the various hallmarks of cancer, the competition for nutrient availability between tumour cells and healthy tissues necessitates metabolic adaptation. Once the first malignant cell evades the homeostatic checkpoints in the organism, it starts a series of changes in the potential tumour and the host, resulting in the survival of the best adapted [5,6,7]. Exploring the different levels of influence where cancer may act, the metabolic adaptations and their consequences can be divided into three categories: metabolic changes that occur (1) internally of the tumour cells, (2) in the tumour microenvironment (TME), and (3) in distal organs resulting in a systemic impact in the host. These three levels of influence can not only affect tumour growth, but also support the metastatic process, one of the systemic hallmarks of cancer [8].

Different questions related to tumour initiation, development, and progression have been addressed using *in vitro* and *in vivo* cancer models, such as cell cultures and animals like *Caenorhabditis elegans*, *Drosophila melanogaster*, *Danio rerio*, and *Mus musculus* [9,10,11,12]. Several genetically engineered animal models have been developed to more accurately study sporadic human cancers by specifically controlling the timing and location of mutations, maintaining the immune systems intact, and providing tumour contexts that are tractable and precise assay for drugs and other interventions *in vivo* [13]. For example, like *D. melanogaster* models, genetically engineered mice are particularly relevant for examining physiological contexts for cancer initiation and adaptive progression towards full-blown tumours, revealing the effect of innate immune response and metabolism in sustaining/restraining cancer initiation and progression. These models include syngeneic mouse models, carcinogen-induced models, and patient-derived xenografts in humanised mice, which enable the study of cancer without compromising immune competence or other physiological aspects [14]. Nevertheless, the short life cycle of *D. melanogaster*, together with the availability of advanced and sophisticated genetic tools, make the fly model exceptional for large-scale studies and the single-cell resolution of cancer initiation, progression, internal and systemic interactions, and the in-depth dissection of the inter-organ communication in cancer [15,16,17,18,19]. The ease of manipulating *D. melanogaster* organs and processes in a temporal and spatial manner allows researchers to focus on fundamental mechanisms while still drawing insights relevant to more complex models [10,20]. It is noteworthy that cancer was detected and researched in the organism *D. melanogaster* more that hundred years ago [21].

This review highlights the current state of research and technologies using the *D. Melanogaster* simplified cancer model to explore key aspects of tumour metabolism, from the changes that cancer cells undergo to the impact of these adaptations on the entire organism. Furthermore, the application of metabolic sensors, new high-throughput technologies (transcriptomics, metabolomics, proteomics, etc.), advanced genetic techniques (dual-system expression), and the use of metabolic modulators in the study of the *D. melanogaster* tumour–host interaction lay the groundwork for new treatments and interventions in human patients.

## 2. Internal Metabolic Tumour Changes

**Figure 1:**
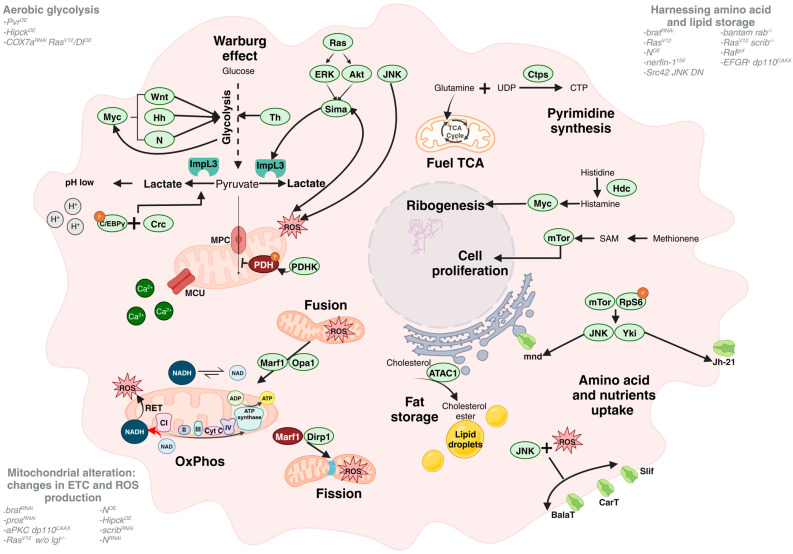
Internal metabolic adaptation in tumour cells. The diagram shows the tumour model genotypes and the processes involved. For aerobic glycolysis: *Pvr^OE^*, *Hipk^OE^*, and *COX7a^RNAi^ Ras^V12^/Dl^OE^* cancer models support the production of lactate via ImpL3, inhibiting the entrance of pyruvate into the mitochondria. In mitochondrial dysfunction: *brat ^RNAi^*, *pros^RNAi^*, *aPKC-dp110^CAAX^*, *Ras^V12^* w/o *lgl^−/−^*, *N^OE^*, *Hipck^OE^*, *scrib^RNAi^*, and *N^RNAi^* models generally require OxPhos to grow through mitochondrial dynamic changes as ETC and ROS production. Furthermore, other dynamic mitochondrial processes and ETC factors are necessary to keep ROS and NADH/NAD ratios. For harnessing amino acid and lipid storage: *brat^RNAi^*, *Ras^V12^*, *nerfin-1^159^*, *N^OE^*, *Src42ACE JNK DN*, *bantam^OE^ rab^−/−^*, *Ras^V12^ scrib^−/−^*, *Raf^gof^*, and *EFGR^λ^ dp110^CAAX^* strains regulate the use of specific amino acids to fuel the TCA cycle, enhance pyrimidine synthesis, support ribogenesis, and drive cell proliferation, alongside the overexpression of amino acid transporters and increased fat storage.

At the first level of cancer organisation, the changes inside a tumoural cell are well-documented; these include genetic mutations, genome instability, replicative immortality, the dysregulation of cell metabolism, etc. [4]. The latter acquires more importance concerning the others because, depending on how the cancer cell manages its nutritional resources, it will have more success in developing tumour mass and even to colonise distant organs (Figure 1). At the tumour niche, oxygen and nutrients are limited, so adapting the metabolism of the tumour cells is a crucial strategy to keep growing and expanding.

### 2.1. Aerobic Glycolysis—Warburg Effect

One of the most studied metabolic adaptations in cancer is “the Warburg effect”, also called aerobic glycolysis, where the oxidative phosphorylation (OxPhos) is diminished to prioritise glycolysis even in the presence of oxygen [22]. Lactate dehydrogenase (LDH) is the enzyme responsible for metabolising the pyruvate, which is the result of the last step of glycolysis, to convert it into lactate. In *D. melanogaster*, LDH is encoded by the *ImpL3* gene, and its upregulation in the tumoural context comes from different routes such as receptors, kinases, or mitochondrial components.

*ImpL3* is upregulated along with other glycolytic genes in epithelial neoplastic tumours when the PDGF/VEGF receptor (Pvr) is overexpressed (*Pvr^OE^*). The connection between Pvr and ImpL3 is mediated by Ras, which activates the extracellular-signal-regulated kinase (ERK) and Akt pathways, leading to the stabilisation of Hypoxia-inducible factor-1α (Hif-1α), known as Sima in *D. melanogaster*, and inducing ImpL3 expression. Moreover, *Pvr^OE^* triggers the phosphorylation of pyruvate dehydrogenase (PDH) by PDHK, blocking the conversion of pyruvate to acetyl-coenzyme A (CoA), which downregulates mitochondrial genes and increases reactive oxygen species (ROS) production via the JNK pathway. This creates a feedback loop that sustains Sima and JNK activity, promoting energy production through aerobic glycolysis [23]. Additionally, further studies with this model have shown the implication of thin (tn) protein in glycolysis management to promote tumour growth [24].

In a wing disc tumoural model, the overexpression of Homeodomain-interacting protein kinase (*Hipk^OE^*) induces the upregulation of Myc, which targets different pathways like Wingless (Wnt), Hedgehog (Hh), and Notch (N). As a result, the level of expression of glycolytic enzymes such as ImpL3, Pgi, HexA-C, and PFK-2 is increased. The last one generates fructose-2,6-bisphosphate (F2,6BP), a potent allosteric molecule that binds PFK-1 to boost glycolysis. Both enzymes interact with Myc to support glycolysis in tumour progression [25]. Furthermore, to avoid proteasomal degradation and protect the tumour, Hipk is coupled to glucose through O-GlcNAcylation [26].

Destabilising the electron transport chain (ETC) by silencing cytochrome c oxidase subunit 7a (*COX7a^RNAi^*) or other close mitochondrial components provokes overgrowth in hyperplastic Delta overexpression (*Dl^OE^*) and active form of Ras (*Ras^V12^*) tumour eye discs. The interaction is mediated by the protein-folding stress factor Cryptocephal (Crc), along with C/EBPγ phosphorylated. Crc is crucial for maintaining the tumour phenotype and targeting glycolytic genes such as *ImpL3*, which, in cooperation with *Dl^OE^*, promote proliferation and overgrowth, enhancing the Warburg effect. Consequently, pH is lower in the cytosol, keeping the tumour folds [27].

ImpL3 has been proposed as a good marker of the Warburg effect, and recent studies have discovered an essential relationship between the aggressiveness of tumours and the upregulation of this enzyme, establishing low levels for hyperplastic and high levels for neoplastic tumours [28,29]. It is necessary to highlight the critical contribution of aerobic glycolysis in models such as the *N^RNAi^* in *D. melanogaster* intestinal stem cells. In this model, the silencing of *ImpL3^RNAi^* or overexpressing the mitochondrial pyruvate carrier (*MPC^OE^*) considerably reduced the number of tumour cell clones [30]. Furthermore, another way to decrease OxPhos is through the inactivation of Mito[Ca^2+^] Uniporter (MCU), which cannot import Ca^2+^ to the inner mitochondrial required by the ETC. This enhances a Warburg-like state that relies on a high NADH/NAD ratio, low ROS, and the restoration of ATP/ADP ratios [31].

### 2.2. Mitochondrial Alteration: Changes in ETC and ROS Production

As mentioned before, aerobic glycolysis is predominant in most cancers, and apparently, the downstream effects of this process, such as the mitochondrial function, lose relevance in the tumoural context. Far from it, different *D. melanogaster* brain tumour models can be highlighted, such as the downregulation of *prospero* (*pros^RNAi^*) or brain tumour (*brat^RNAi^*) and cooperation between atypical protein kinase C (aPKC) and the active form of the PI3K catalytic subunit dp110 (*aPKC-dp110^CAAX^*), which require OxPhos to sustain and grow [32]. This also seems to occur in *lethal giant larvae* mutant (*lgl^−/−^)* tumours clones (alone or combined with *Ras^V12^*), where the silencing of the NADH dehydrogenase NDUFV1 (*NDUFV1^RNAi^*), a component of respiratory Complex I (CI), forces glucose consumption and lactate production, reducing tumourigenic potential and proliferation. Furthermore, this silencing prevents the stabilisation of Sima, reducing invasion via Matrix metalloproteinase 1 (Mmp1) [33]. CI is the first enzymatic complex of the ETC rate-limiting of OxPhos and is involved in a poorly described phenomenon called reverse electron transport (RET). The transference of electrons from NADH by NDUFV1 to ubiquinone through several Fe-S clusters is reverted, producing high ROS and a low NAD/NADH ratio. *N^OE^* in the brain model and, probably, in gut tumours is behind this change in electron flow, but the mechanism is unknown [34].

At a structural level, it is well known that mitochondria are dynamic organelles that depend on the cell’s energy demand. These demands can be dampened by fusion to produce more energy or fission to decrease the energetic yield. In the tumoural context, mitochondrial fusion mediated by the Dynamin-like GTPases Marf and Opa1 [35] has been described to increase OxPhos, ROS, and NAD/NADH ratios, as happens in immortalised malignant *brat^RNAi^* brain cells [36] and wing disc *Hipck^OE^* tumour-like cells [37]. In this case, glycolytic enzymes such as PFK-2 may also regulate this process [37]. By contrast, there are cases of the inhibition of *scribble* (*scrib^RNAi^*) wing disc tumours, where high ROS come from the fission process managed by Dirp-1 [38].

ROS are a side effect of dynamic mitochondrial organisation, but other ways exist to alter it. Other features of ROS rely on initiating an invasive process to metastasis, as will be discussed in depth below. Briefly, the deregulation of some oncogenes can raise tumours with high ROS but without invasion capacity. Only in combination with other inhibited factors in the ETC, such as CI (*Psdw*) and ATPsynβ in *Hipck^OE^* tumours [37] or by extracellular matrix-deprivation (*mys^RNAi^*) in *N^RNAi^* ISC tumours [39], is ROS enhanced and the invasive transition initiated.

### 2.3. Harnessing Amino Acids and Lipid Storage

Amino acids are crucial elements for the synthesis of proteins, nucleic acids, and lipids, which are vital for cancer cell proliferation. Although glutamine is a non-essential amino acid, it is necessary for the survival of many tumour cells. It has been involved in many processes as a primary energy source to fuel the tricarboxylic acid (TCA) cycle, promoting OxPhos to sustain *brat^RNAi^* tumour growth [36]. Furthermore, in *Ras^V12^* malignant cells, glutamine and uridine triphosphate (UTP) substrates are needed for glutamine synthase, called CTP synthase (Ctps), to produce cytidine triphosphate (CTP) for pyrimidine synthesis, an essential step for tumourigenesis [40]. Another amino acid to highlight is histidine. In models of neural cancer stem cells such as nervous fingers 1 (*nerfin-1^159^*) null mutant clones and *N^OE^*, a predicted histidine decarboxylase (Hdc) catalyses the conversion of L-histidine into histamine, modulating Myc to enhance ribogenesis and promoting tumour development [41]. Finally, in the constitutively active form of Src42A and the dominant-negative form of JNK (*Src42A CA*, *JNK DN*) wing disc tumour model, methionine is converted into S-adenosylmethionine (SAM), which then activates the TOR signalling pathway, leading to increased cell proliferation [42]. So far, little research has described how different amino acid transporters and internal nutrient sensors can manage tumour progression. An example of this has been described in the *Ras^V12^scrib^−/−^* or the new eye disc tumour model, which is based on the oncogenic cooperation between the overactivation of a pro-growth microRNA *bantam* and knockout of small GTPase involved in producing early endosomes (*bantam^OE^ rab5^−/−^*). Two amino acid transporters involved in the tumour’s growth were highlighted: Juvenile Hormone-21 (Jh-21) and minidisc (mnd), with Jh-21 downregulation exhibiting a strong decrease in the tumour phenotype. JNK and yorkie (yki) mediate their upregulation through the activation of the nutrient sensor mTOR and phosphorylation of ribosomal protein S6 (RpS6) to promote tumour growth and invasion [43]. Slim-fast (Slif), CarT, and BalaT are transporters involved in the import of amino acids and their derivatives like carcinine, histamine, and β-alanine. Their silencing in Raf-gain of function (*Raf^gof^*) gut tumours decreased its growth [44].

Not only amino acids and glucose pathways sustain tumour growth; lipid metabolism and the related enzymes seem to play a role in the tumourigenic process. In *EGFR^λ^ dp110^CAAX^* glioma cells, a key regulator of cholesterol metabolism in the endoplasmic reticulum, acyl-CoA: cholesterol acyltransferase 1 (ACAT1) converts cholesterol into cholesterol esters for storage as lipid droplets [45].

In cancer models, *D. melanogaster* tumours often exhibit metabolic reprogramming similar to the Warburg effect seen in mammals, where there is an increased reliance on glycolysis for rapid energy production. However, research on *D. melanogaster* cancer metabolism has primarily focused on glycolysis and mitochondrial functions, with less emphasis on the roles of other amino acids, the synthesis of nucleic acids, and the mobilisation and use of lipids [46]. These macromolecules are crucial for cell growth, signalling, and maintaining the structural integrity of cells, yet their specific contributions to tumourigenesis in *D. melanogaster* remain underexplored. This gap in knowledge is significant because different tumours may have unique metabolic needs, suggesting that *D. melanogaster* models could provide valuable insights into tumour-specific metabolic adaptations that are not fully understood in other systems. Further research into these areas could enhance our understanding of how metabolic pathways support cancer progression in *D. melanogaster* and their relevance to human disease.

## 3. Metabolic Changes in Tumour Microenvironment

**Figure 2:**
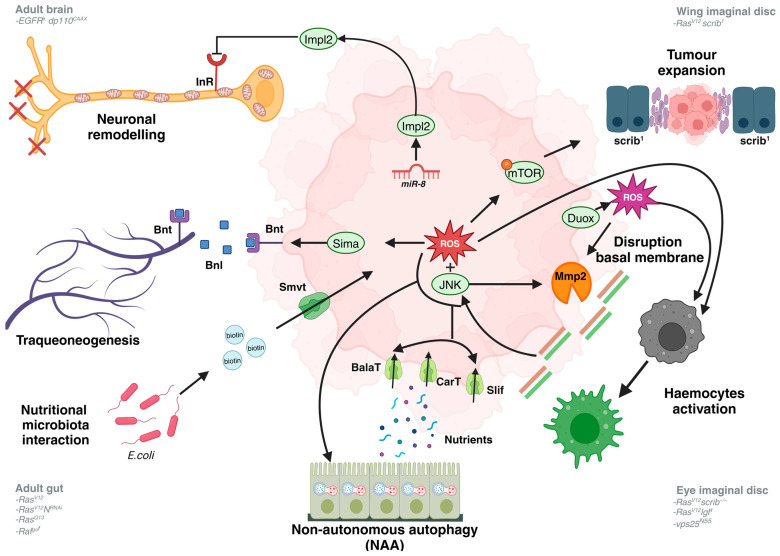
Metabolic interaction between tumour cells and their microenvironment. The diagram displays the main processes between tumour and neighbouring cells, which rely on different larval and adult tumour models. Adult tumour brain by *EGFR*^λ^ *dp110^CAAX^* mediates neuronal remodelling. Adult tumour gut by *Ras^V12^*, *Ras^V12^ N^RNAi^*, *Ras^Q13^*, and *Raf^gof^* manage different processes such as tracheoneogenesis, nutrition microbiota interaction, and non-autonomous autophagy. Mutation in epithelial imaginal discs as wing (*Ras^V12^ scrib^1^*) and eye (*Ras^V12^ scrib^−/−^*, *Ras^V12^ lgl^4^*, *vps25^N55^*) support tumour expansion and haemocytes activation via disruption of BM, respectively.

Many of the changes in tumoural cells arise from their interaction with neighbouring healthy cells. This section will describe the non-autonomous effects produced by the oncogenic cooperation within cancer cells, which impact their microenvironment (Figure 2). These effects include metabolic adaptations that depend on the tumour’s immediate neighbours, such as neurons, immune cells, or epithelial cells. The following section will explore the metabolic changes in distal cells or organs far from the tumour. It is important to note that some tissues do not have a clear physical boundary to distinguish whether the interaction concerns the microenvironment or systemic changes.

A *D. melanogaster* model of glioblastoma that combinates the constitutively active forms of EGFR and PI3K (*EGFR^λ^*, *dp110^CAAX^*) demonstrates that secreted imaginal morphogenesis protein-late 2 (ImpL2) through the activation of *miR-8* from the glial-derived tumoural cells antagonises insulin signalling in neighbouring neurons. This antagonism induced mitochondrial disruption and synapse number reduction, ultimately promoting neurodegeneration [47]. There is evidence showing that adult intestinal tumours (*Ras^V12^*, *N^RNAi^* or *Ras^Q13^*) induce tracheoneogenesis, a process reminiscent of neoangiogenesis, a well-known feature of mammalian cancer [4]. In *D. melanogaster*, the trachea is the functional equivalent of mammalian blood vessels. ROS stabilises Sima promoting Bnl/Bnt signalling, and gut tumours increase the terminal branching of the associated trachea, inducing intestinal stem cell proliferation and increasing oxygenation, promoting tumour growth [48].

Furthermore, in *Ras^Q13^* tumours, there is a nutritional microbiota interaction where bacteria such as *E. coli* release biotin through the upregulation of Smvt, which controls tumour growth [49]. Another way gut tumours (*Raf^gof^*) obtain nutrients is through the neighbours’ cells by activating amino acid transporters, as described before [44], and inducing non-autonomous autophagy (NAA). This phenomenon occurs due to excessive ROS present in cancer cells via the upregulation of JNK, which induces autophagy in the closer cells, releasing an amount of nutrients picked up by the tumour to keep growing [44].

The Bergmann group also described how mitochondrial and extracellular ROS by Duox, an NAD(P)H oxidase, could mediate interactions through the activation and recruitment of the immune system [50]. Furthermore, a correlation exists between neoplastic models such *Ras^V12^ lgl^4^* and *vps25^N5^*^5^ that showed high ROS with a basal membrane (BM) disruption via the activation of Mmp2 to promote invasion. That resulted in the upregulation of JNK and the recruitment of haemocytes to activate them. By contrast, hyperplastic models such as *hippo* and *eyeful* showed no BM damage either recruitment [51]. Mitochondrial ROS can modulate cell competition in *Ras^V12^ scrib^1^* clones in *scrib^1^* background neighbour cells. These tumour clones provoke expansion and apoptosis in surrounding cells by activating the nutrient sensor mTOR [52].

## 4. Systemic Metabolic Effects

**Figure 3:**
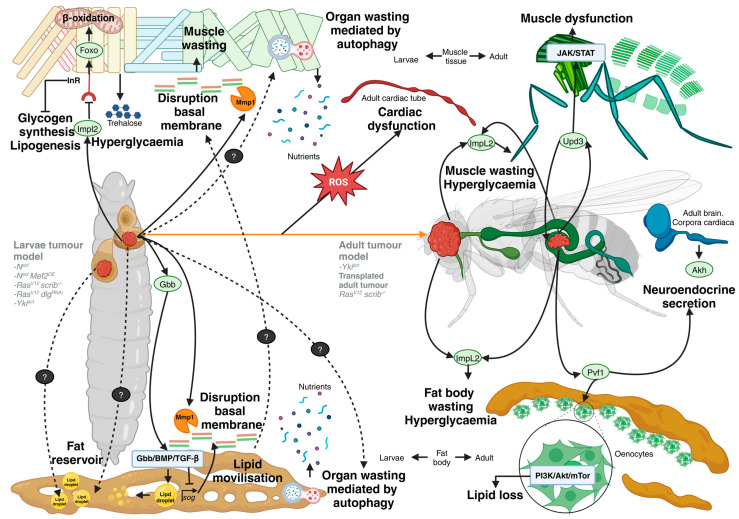
Systemic metabolic interactions. The diagram shows the different systemic metabolic adaptations in larval and adult tumour models. Imaginal disc tumours originated by *N^act^*, *N^act^ Mef2^OE^*, *Ras^V12^ scrib^−/−^*, *Ras^V12^ dlg^RNAi^*, or *Yki^act^* interact mainly with muscle tissues and fat body. The first one is to inhibit glycogen synthesis and lipogenesis, generate hyperglycaemia, and disrupt BM, provoking muscle wasting. In the fat body, there is an increase in fat reservoir and, by contrast, an increase in lipid mobilisation and the disruption of the basal membrane. In both tissues, nutrients are released by an increase in autophagy. Adult tumour tissues as gut by *Yki^act^* or transplantation of imaginal discs *Ras^V12^scrib^−/−^* provoke muscle and fat body wasting via ImpL2 secretion. Furthermore, *Yki^act^* induces lipid loss in the oenocytes, neuroendocrine secretion, and muscle dysfunction. Tumour induction in the eye imaginal disc generates cardiac dysfunction in the adult fly.

Interactions between multiple organs are essential for maintaining proper physiological functions and understanding the pathology of various diseases. Many organs are physically separated and communicate through blood and lymph circulation via diverse signals, such as soluble factors, hormones, exosomes, cells, and neural innervation. Organs rely on nutrients from the diet and must convey their needs to organs responsible for processing and storing these nutrients. This communication involves well-established soluble factors like cytokines, hormones, and other unknown factors that may be altered in disease contexts such as cancer. Additionally, systemic communication includes feedback loops and bi-directional or multi-directional interactions [20,53]. *In vivo* studies in whole organisms, like *D. melanogaster*, are crucial for unravelling the temporal processes and inter-organ communication that regulate homeostasis and its disruption during the initiation and progression of cancer (Figure 3).

Cancer cells can alter systemic metabolism to enhance the release of energy and nutrients from body storage, achieving the demands of cancer cell proliferation. A metabolic imbalance in the host triggered by the tumour produces cachexia, characterised by a general state of weight and muscle loss, which is a primary cause of death in cancer patients [54]. Several *D. melanogaster* tumour cachexia models contributed to identifying tumour-derived factors that influence systemic metabolism and tissue wasting. Two adult tumour models with muscle wasting phenotypes, such as *Yki^act^* and *Ras^V12^scrib^−/−^*, identified the ImpL2 as a tumour-derived cachectic factor [55,56]. High levels of ImpL2 in the haemolymph reduce systemic insulin/IGF signalling, inhibiting glycogen synthesis and lipogenesis, ultimately causing organ wasting. Both cachectic adult tumour models exhibit hyperglycaemia (increased trehalose, a kind of blood sugar) due to the systemic reduction in insulin signalling by tumour-derived ImpL2. In the *Ras^V12^scrib^−/−^* and *Ras^V12^dlg^RNAi^* tumour models in the larval developing eye, the tumour-secreted ImpL2 systemically decreases insulin signalling, altering the activity of the transcription factor FOXO in the muscle. Downstream of FOXO, mitochondrial fusion and increased β-oxidation led to increased muscle lipid utilisation [57]. Enhanced autophagy also drives muscle wasting in the *Ras^V12^ scrib^−/−^* model [58].

In the adult *Yki^act^* gut tumour model, tumour-secreted factors Pvf1 and Upd3 also contribute to organ wasting. Pvf1 induces lipid loss by stimulating Pi3K/Akt/mTOR signalling in oenocytes (analogous to mammalian hepatocytes), mobilising lipid storage and triggering the secretion of adipokinetic hormone (Akh), a glucagon-like catabolic hormone, from neuroendocrine cells [59]. Upd3 causes wasting by activating the JAK/STAT signalling pathway in host organs [60]. Glass bottom boat (Gbb) secreted from the tumour in the *Ras^v12^dlg^RNAi^* larval model activates Gbb/BMP/TGF-β signalling in the fat body the *D. melanogaster* equivalent of mammalian adipose tissue, and in the muscle, leading to increased lipid mobilisation [61]. High levels of tumour-derived Mmp1, observed in most *D. melanogaster* cachexia models, lead to fat body and muscle degeneration. Mmp1 disrupts the fat body by downregulating TFG-β signalling and the muscle BM, altering the localisation of extracellular matrix (EMC) proteins, where TFG-β antagonist short gastrulation (sog) and insulin signalling converge in the fat body to regulate ECM protein secretion [61,62].

Cardiac dysfunction in cancer patients is generally associated with cardiotoxicity induced by therapeutic agents used to treat cancers and/or cancer-induced cachexia. Whether tumours systemically affect heart function before treatment and/or the onset of cachexia is still unknown. More recently, increasing evidence suggests that the associated systemic cardiac dysfunction could directly affect tumour biology. The larval *Yki^ac^*^t^ tumour model proposes a mechanism involved in cancer-mediated heart failure. Tumour growth in the imaginal eye disc causes a systemic increase in ROS, contributing to increased oxidative stress in adult cardiac tube and compromising cardiac function [63].

Altered lipid metabolism is a common tumour-triggered host response, and increased lipid synthesis facilitates cancer progression. The inhibition of enzymes involved in lipid biosynthesis has been shown to block cancer growth [64]. *N^act^* in the wing imaginal disc and *N^act^* combined with the overexpression of Myocyte enhancer factor 2 (*N^act^ Mef2^OE^*) in the eye imaginal disc led to the significant modulation of lipid metabolism. This is evidenced by increased lipid droplet size in the fat body, which serves as an energy reservoir for the host. Consequently, the gene expression involved in lipolysis and lipogenesis is altered in the tumour-bearing host [65].

The same authors also demonstrated that lipid droplets affect the innate immune response. Systemic infection in adult flies results in lipid droplet accumulation in the midgut, correlating with increased whole-body lipid levels and the activation of immune signalling pathways. These findings establish lipid droplet accumulation as a marker and regulator of the antibacterial immune response [66]. Other studies support the lipid–immune interactions, for example, the activation of Toll signalling in the larval fat body induces significant changes in lipid metabolism, enhancing immune function while reducing lipid storage [67]. Lipin and Midway (Mdy) proteins act as key regulators of lipid storage, facilitating the conversion of fatty acyl-CoA to triglycerides (TG). Meanwhile, the perilipin family of proteins modulates lipolysis rates, making TG more accessible for metabolic needs across tissues. Lipid droplets play an important and dynamic role in balancing lipid degradation and synthesis [66]. Interestingly, perilipins respond to immune signals by modifying lipid droplet structure to help alleviate inflammatory stress [68]. The reorganisation of lipid droplets presents a potential therapeutic target for resolving inflammation; it would be interesting to elucidate the interrelationship between innate immunity and lipid metabolism in the context of cancer.

In the larval *Ras^V12^scrib^−/−^*, increased serum levels of amino acids such as arginine, lysine, and proline have been reported due to systemic elevated autophagy [58]. However, how the tumour exploits extracellular amino acids remains to be characterised. Newton and colleagues uncovered a tumour-promoting role of proline in response to systemic host metabolic changes using an obesity-enhanced tumour model that combines RasV12 with the knockout of the negative regulator of Src, C-terminal src (*Ras^V12^csk^−/−^*) raised on a high-sugar diet (HSD) [69].

Finally, metabolic diseases are also associated with increased cancer incidence. In *scrib^−/−^* mutant cells, hyperinsulinemia (high levels of circulating insulin) systematically abrogates tumour-suppressive cell competition by boosting InR-mTOR-mediated protein synthesis in premalignant cells [70].

## 5. Metabolic Adaptations in Metastatic Tumours

Each phase of metastasis reflects the ability of tumoural cells to evade the host’s immune system and adapt to new microenvironments thanks to their phenotypic plasticity. Cancer cells acquire metastatic traits through genetic mutations and inherent predispositions within the tumour cell population [71]. Metabolism is crucial in these cellular transitions, as cancer cells require constant energy and nutrients to proliferate, migrate, and form new tumours [72]. Metabolic rewiring in metastatic cells can occur through transcriptional control, epigenetic modifications, post-translational modifications, and metabolite availability to enzymes [72,73]. These adaptations, outstanding to *D. melanogaster*, may be crucial in understanding and potentially targeting human metastatic tumours. Here, we present the most recent advances relating to cancer metastasis and metabolic adaptations discovered in *D. melanogaster* models.

Aldehyde dehydrogenase (ALDH) is a detoxifying enzyme that has essential functions in developing epithelial homeostasis and may play a role in forming distant bone metastases [74]. A study using a *D. melanogaster in vivo* metastasis model provided evidence that the effects of ALDH7A1 on cell migration and invasive behaviours are mediated by a decrease in the levels of metabolites that function as ligands for the peroxisome proliferator-activated receptor (PPARα) [75]. The study found that ALDH7A1 activity can influence various metabolic pathways and cellular functions, potentially impacting disease progression. These findings suggest that patients with low ALDH7A1 levels may benefit from therapeutic approaches that target the activity of PPARα [75].

A very recent study investigated the long-term effects of perfluorooctanoic acid (PFOA) exposure in *D. melanogaster* over three consecutive generations. The findings from this study are concerning, as they suggest a substantial multigenerational metastatic risk associated with prolonged PFOA exposure. The study found that PFOA exposure significantly promoted tumour invasion rates in the *D. melanogaster* model and disturbed the activities and content of fatty acid synthase, acetyl-CoA carboxylase, and the protein binding to sterol regulatory elements. These metabolic changes indicate that PFOA exposure can induce substantial metabolic reprogramming in cells, possibly contributing to the increased metastatic potential observed [76]. Notably, the detrimental effects of PFOA exposure persisted across three consecutive generations of *D. melanogaster*, highlighting the potential for long-term multigenerational impacts on metastatic risk. These findings underscore the importance of understanding the complex interactions between metabolism, antioxidant responses, and rhythm regulation in the context of environmental exposures and their consequences for cancer metastasis.

MMPs are key players that facilitate the local invasion and infiltration of the invasive front of tumours. The endothelial BM is a physical barrier that prevents cancer cells from infiltrating the surrounding stroma and extravasating to distant organs after initial engraftment [77]. MMPs play a crucial role in degrading and remodelling the BM, allowing cancer cells to overcome this barrier and invade the neighbouring tissue. Wei. T and colleagues demonstrated, with the help of the *D. melanogaster* malignant tumour models, *Raf^gof^ scrib^−/−^* and *Ras^V12^ lgl^−/−^*, that the tumour overgrowth, invasion, and distant metastasis could be inhibited by *foi* knockdown in tumour clones. They provided evidence that intracellular zinc regulates the expression of MMPs and their activities *in vivo* [78]. Interestingly, *foi* is a homolog to mammalian ZIP6 and ZIP10, highly expressed in several breast cancers, and associated with cancer invasion and metastasis. Additionally, cells expressing plasma-membrane-located ZIP6 undergo EMT and detach from monolayers as highly proliferating cells [79]. This study links zinc metabolism and the action of MMPs during EMT to promote metastasis.

M2-like tumour-associated macrophages (TAMs) are commonly found in the tumour microenvironment and are associated with pro-angiogenic, immunosuppressive, and pro-metastatic functions [77]. A recent study identifies Atossa (*Atos*) as a crucial regulator of energy levels in *D. melanogaster* macrophages, showing increased mRNA levels before tissue invasion and sustained elevation during the process. *Atos* enhances mRNA levels of Porthos, a DEAD-box protein, and of two metabolic enzymes, lysine-α-ketoglutarate reductase and NADPH glyoxylate reductase, thus enhancing mitochondrial bioenergetics, which is essential for invasion. The *in vivo* findings of this study align with previous *in vitro* works, which showed that higher ATP levels are needed for the first cancer cell to migrate through the extracellular matrix [80]. However, this study also identifies a specific molecular pathway centred around the Atossa protein that can produce the higher energy levels required for challenging cellular tasks like tissue invasion [81].

Metastasis suppressor genes (MSGs) also play a crucial role in inhibiting specific biological processes during metastatic progression without globally influencing primary tumour development. One of the first identified MSGs in *D. melanogaster* is abnormal wing discs (*awd*). This group of genes encodes enzymes with highly conserved nucleoside diphosphate kinase (NDPK) activity, which has been extensively studied [82]. The findings on NDPKs in model organisms, such as *D. melanogaster*, have converged on our understanding of the human NM23 proteins, particularly NM23-H1, and their roles within the metastatic cascade. Studies in *D. melanogaster* revealed that *awd* acts as a negative regulator of cell motility through the endocytosis of chemotactic receptors on the surface of migrating cells [83]. Consequentially, these discoveries have led to the formulation of possible targets for metastasis, such as the study of Lee et al. (2018) that reported the discovery of a small molecule NDPK activator that was named NMac1, which acts as a metastasis suppressor by activating NM23-H1. Moreover, this study suggests using NMac1 in combination therapy with anti-tumour agents in breast cancer [84].

As explained in the internal tumoural metabolic changes section, ImpL3 plays an essential role in the Warburg effect. It looks to have more than one process in the tumour development inside and outside cancer cells. Furthermore, it acts as a cooperating factor essential for epidermal growth factor receptor (EGFR)-driven epithelial neoplasia and metastasis. Genetic modifications that enhance glucose metabolism, along with HSD, were found to promote EGFR-driven neoplasia, with this process being dependent on ImpL3. These results suggest that the Warburg effect on metabolism is more significant in driving neoplasia and metastasis than previously recognised [85].

Cachexia has been observed in larval tumour models, allowing investigation into links with tumour invasion and metastasis. A study using the metastatic *Ras^G12V^csk*^−/−^ *D. melanogaster* model revealed a systemic amino acid-utilising circuit whereby HSD-enhanced tumours with secondary tumour formation induce muscle wasting and the release of proline into the circulation. The study also highlights the crucial role of the SLC36-family amino acid transporter Path in tumour growth and reveals a proline vulnerability. Furthermore, the study demonstrated that exogenous proline fosters tumourigenesis through Path, revealing a dual-layered coordination in the tumour’s metabolic response. Tumours induce muscle wasting at the whole-organism level and boost systemic amino acid availability. On the tumour-autonomous level, tumours adapt their amino acid transporter repertoire to leverage the increased amino acid availability [69].

In the same line, using Computed Tomography, it was observed that *Ras^V12^ scrib*^−/−^ tumours grow 10-fold when invading the VNC while exhibiting a 50% reduction in muscle volume compared to the benign *Ras^V12^* control. Also, a mass spectrometry assay detected increased circulating sugars and amino acids as autophagy progressed. These findings underscore the potential of targeting autophagy for pharmacological intervention to mitigate metastasis-associated mortality and morbidity [58].

Overall, *D. melanogaster* models have provided valuable insights into the role of metabolism in cancer, including metastasis. However, further research is needed to fully elucidate the metabolic adaptations and vulnerabilities that arise during the metastatic process.

## 6. New Technologies to Explore Internal and External Metabolic Tumour Changes

*D. melanogaster* has been extensively utilised as a powerful genetic model system, with various genetic tools available for manipulation and study. Having shown the metabolic changes across three levels of cancer organisation—intracellular, tumour microenvironment, and systemic levels—we aim to introduce the new technologies developed over recent years. Utilising diverse approaches to analyse alterations in metabolic pathways [86] within the tumoural context, we intend to provide comprehensive insights at the metabolic, genetic, and proteomic levels, emphasising key techniques in studying cancer metabolism within the *D. melanogaster* model system.

### 6.1. Metabolism Sensor Toolkit

Metabolites, unlike other macromolecules such as proteins, RNA/DNA, or polysaccharides, can undergo rapid modification, transformation, and degradation. Therefore, precise analytical tools are required to accurately identify the metabolic states at specific time points, enabling us to discern changes associated with the disease or mutant under investigation (Table 1).

Genetic sensors coupled with the GAL4/UAS system allow for the temporal monitoring of specific metabolites. By utilising Förster resonance energy transfer (FRET) technology, a reduction in the emission spectrum is observed when metabolites bind to the sensors, facilitating the detection of these changes. For example, Wong et al. (2019) and Gandara et al. (2019) used these sensors to demonstrate that alterations in the levels of metabolites associated with the Warburg effect occur within the tumour context, including glucose (the central energy source), lactate (Laconic), pyruvate (Pyronic), and 2-oxoglutarate (OGsor) [25,28].

Downstream of aerobic glycolysis, various methods are available to detect mitochondrial disruptions, such as changes in the NADH/NAD or ATP/ADP ratios. For instance, Sonar and PercivalHR are dual excitation/single emission sensors that exhibit distinct emission spectra shifts depending on the bound molecule [31]. Other mono-fluorescent sensors linked to GFP can detect redox reactions, such as H_2_O_2_ with gstD-GFP [23] and MitoRoGFP2_Orp1 and glutathione oxidation with MitoRoGFP2_Grx1 [31]. Additionally, a known consequence of aerobic glycolysis is a decrease in pH within the tumour environment, which is measurable using pH sensors [27].

An alternative method for tracking glucose uptake involves the use of fluorescent analogues recognised by glucose transporters and hexokinase. One such analogue, 2-deoxy-2-[(7-nitro-2,1,3-benzoxadiazol-4-yl)amino]-D-glucose (2-NBDG), has been employed to monitor glucose uptake in tumour contexts [25].

### 6.2. Omics Technologies in the Metabolic Tumoural Context

Omics technologies have revolutionised our understanding of cancer metabolism, providing unprecedented insights into the complex metabolic reprogramming that occurs in tumours. Here is an overview of how various omics approaches are being applied to study metabolic alterations in cancer (Table 2).

#### 6.2.1. Metabolomics

To detect a broader range of metabolites rather than being restricted to just one or a few, high-throughput methods such as omics technologies have been developed, allowing for the detection and quantification of hundreds of metabolites, a field known as metabolomics. The primary tools used in metabolomics include gas chromatography (GC), liquid chromatography (LC), capillary electrophoresis (CE), and nuclear magnetic resonance (NMR) coupled with mass spectrometry (MS). Depending on the approach, metabolomics can be “untargeted”, where all detectable molecules are analysed, or “targeted”, focusing on specific known metabolites and pathways. This technique has been applied across various fields, including development, ageing, temperature stress, oxygen sensitivity, and nutrition [87,88].

Recent studies in metabolomics using LC-MS have followed a standard methodology involving the dissection and homogenisation of tissues such as whole brains, thoraces, larval wing discs, and haemolymph; solvent extraction; chromatographic separation with reverse-phase columns; and MS analysis employing electrospray ionisation in both positive and negative ion modes. Specific ion transitions, multiple reaction monitoring (MRM), and parallel reaction monitoring (PRM) have been used for precise detection and quantification, with data analysis facilitated by software such as MassLynx^TM^. For instance, Kwon et al. (2015) investigated metabolites altered by organ wasting in *yki^act^* flies, finding decreased levels of ATP and NADH/NADPH, and increased levels of trehalose, indicating reduced whole-body triglycerides and glycogen [56]. Bonnay et al. (2020) combined transcriptomic data with targeted metabolomics, uncovering elevated TCA cycle intermediates in *brat^RNAi^* brain tumours and identifying glutamine as a key energy source [36]. Newton et al. (2020) reported elevated levels of free amino acids, such as L-proline in haemolymph, in *ras1^G12V^ csk^−/−^* tumours, highlighting proline’s role in tumour growth [69]. Nishida et al. (2021) quantified methionine and SAM in larval wing discs and haemolymph [42], while Khezri et al. (2021) performed untargeted metabolomics to detect changes in haemolymph macronutrients in *Ras^V12^scrib^−/−^* contexts, revealing significant metabolic reprogramming associated with cancer [58].

The targeted metabolomics of haemolymph amino acids has been conducted to detect the increase in free amino acids and 3-methylhistidine, a biomarker for muscle atrophy, in the *ras1^G12V^ csk^−/−^* tumour context. Haemolymph was pooled from larvae and analysed using capillary electrophoresis coupled with time-of-flight mass spectrometry (CE-TOF-MS) or triple quadrupole mass spectrometry (CE-QqQ-MS) [69].

Maravat et al. (2021) investigated the *EGFR^λ^ dp110^CAAX^* glioma model, and detected metabolites associated with energetic pathways (glycolysis and the TCA cycle) and anabolic pathways (amino acid metabolism and the TCA cycle), using two complementary NMR techniques: H1 solution-state NMR and High Resolution-Magic Angle Spinning (HR-MAS) NMR. Notably, lipids were absent in the solution-state NMR analysis due to the solvent chosen for extraction. Myo-inositol (MI) emerged as the most prominent marker, suggesting that the model is more representative of low-grade astrocytoma or that the blood–brain barrier remains intact. NMR analysis in this *D. melanogaster* glioblastoma model effectively mimics human glioblastomas and serves as a valuable tool for identifying new biomarkers [89].

In a related study, Froldi et al. (2019) utilised targeted H1-NMR to analyse tumour development in adult *D. melanogaster* flies fed diets with or without histidine. The gut content of the flies was cleared of histidine prior to metabolite extraction. The results showed that the histidine-deficient diet effectively depleted most free histidine and 3-methyl-L-histidine, while histamine was below the detection limit. This depletion influenced the growth of *nerfin-1^159^* clones, suggesting that histidine’s effects on clone growth are mediated by its downstream metabolites, 3-methyl-L-histidine and histamine [41].

#### 6.2.2. Prote- and Lipidomics

Proteomics employs high-throughput techniques to analyse the complete protein content of biological samples and assess post-translational modifications such as phosphorylation, as well as protein–protein interactions. These techniques generally fall into three categories: protein separation, protein identification, and protein quantification. Methods used for protein separation may include 2D gel electrophoresis, while protein identification and quantification often utilise LC-MS, among other gel-based and gel-free techniques. Proteins are enzymatically digested into peptides, which are then separated and analysed by LC-MS. Quantitative proteomics measures protein abundance on both relative and absolute scales, enabling comparisons between samples and providing insights into cellular processes, disease mechanisms, and biomarker discovery [90]. In 2021, Lodge et al. used shotgun proteomics to examine how proteins such as Mmp1 and ImpL2 are secreted into the haemolymph by *Ras^V12^dlg^RNAi^* eye tumour discs, contributing to organ dysfunction and wasting. The resulting peptides were analysed by LC-MS using a linear gradient separation on a column coupled to a hybrid quadrupole mass spectrometer [61]. In a complementary study focused on cancer cachexia, Dark et al. (2024) investigated protein upregulation related to lipid metabolism in muscles from *Ras^V12^ dlg^RNAi^* eye tumour discs. Notably, Whd, a protein involved in regulating the β-oxidation pathway, was upregulated. In this study, the peptides were purified, separated by LC-MS, and the data were processed and quantified using specific ion chromatograms after searching against the *D. melanogaster* database [57].

Lipidomics, similar to proteomics, employs high-throughput methods to analyse lipids in biological samples, focusing on lipid composition, structure, and function. Lipid extraction typically involves solvents such as chloroform and methanol, followed by separation using methods like UPLC-ESI-MS. Lipid identification and quantification are achieved through LC-MS. Quantitative lipidomics measures lipid levels across different samples, offering insights into cellular lipid metabolism, disease mechanisms, and potential lipid-based biomarkers [91]. In a study using *scrib^RNAi^* as a cancer model in *D. melanogaster*, untargeted lipidomics identified specific lipid markers associated with cancer, including GM3 (monosialodihexosylganglioside) and Glucosylceramide (GlcdE), which are implicated in tumour progression [92].

#### 6.2.3. Carbon Transfer Measured by Stable Isotope Ratios (CATSIR)

To investigate how tumours incorporate carbon sources from the host or diet, Holland et al. (2021) developed a novel tool based on differential carbon isotope ratios (C13/C12), expressed as δ13C in per mil (‰) units [93]. This method utilises isotope ratio mass spectrometry (IRMS) for precise measurement. Tumour-bearing larvae, specifically those with eye tumours induced by *Ras^V12^ scrib^−/−^*, were fed diets with varying carbon isotope ratios at different stages of development and tumour growth. After feeding, both tumour and host tissues were dissected and prepared for IRMS analysis. The findings revealed that tumour expansion primarily utilises carbon sources from the host rather than the diet. This technique offers potential for exploring how different nutritional interventions might influence tumour behaviour by altering nutrient uptake from host tissues.

**Table 2:** Omics technologies in *D. melanogaster* cancer metabolism field.

Omics	Technology	Tumour Model	Target Tissue	Reference
Metabolomic	Hybrid triple quadrupole LC-MS in positive/negative switching mode	*yki^act^* gut tumours	Haemolymph	[56]
Targeted metabolomics	Tissue cultured with isotopically labelled substrates and HPLC-MS	*brat^RNAi^* brain tumours	Whole brain	[36]
Targeted metabolomic	CE-TOF-MS and CE-QqQ-MS	*ras1^G12V^csk^−/−^* eye disc tumour	Haemolymph	[69]
Targeted metabolomic	UHPLC—hybrid quadrupole MS/MS	*ras1^G12V^csk^−/−^* eye disc tumour	Haemolymph	[69]
Targeted metabolomic	CE-TOF-MS	*Src42A CA JNK DN* wing disc tumour	Haemolymph	[42]
Targeted metabolomic	UPLCH—QqQ-MS/MS	*Src42A CA JNK DN* wing disc tumour	Haemolymph Wing disc	[42]
Untargeted metabolomic	HPLC—Orbitrap MS	*Ras^V12^scrib^−/−^* eye disc tumour	Haemolymph	[58]
Metabolomic	HR-MAS and H^1^-NMR coupled MS	*EGFRλ^OE^dp110C^AAX^* glial tumour	Brain	[89]
Metabolomic	H^1^-NMR—MS	*nerfin-1^159^and pros^-^* brain tumours	Adult flies	[41]
Proteomics	UHPLC—Orbitrap MS	*Ras^V12^dlg^RNAi^* eye disc tumour	Haemolymph	[61]
Proteomics	LC-MS	*Ras^V12^dlg1^RNAi^* eye disc tumour	Eye disc	[57]
Untargeted lipidomics	UHPLC—MS	*scrib^RNAi^* gut tumour	Gut	[92]
CATSIR	IRMS	*Ras^V12^scrib^−/−^* eye disc tumour	Eye disc and host tissues	[93]

### 6.3. Metabolic Pathways Using Bulk and Single-Cell RNA-Seq Methods

One of the significant technological advancements in recent decades is the specification of transcriptional characterisation at the single-cell level. Traditionally, most biological processes have been elucidated using bulk RNA sequencing (RNA-seq), which provides averaged gene expression profiles from entire tissues or large populations of individual cells. However, this approach fails to capture the heterogeneity and distinct subtypes of cells within tissues. The advent of single-cell RNA sequencing (scRNA-seq), first demonstrated by Tang et al. (2009) in characterising four-cell-stage blastomeres [94], has since been applied to a wide range of tissues across various species, from plants to humans [95]. This technology allows for the more detailed profiling of individual cells under wild-type conditions and in various contexts such as gene knockouts, diseases, cancers, disorders, and syndromes, thereby providing deeper insights into cellular diversity and function.

The majority of these studies have been conducted in murine models and humans. However, *D. melanogaster* is also extensively used in various research fields, including immunology, neuroscience, development, and cancer. Numerous studies have been performed on different tissues such as the brain, gut, imaginal discs, and across various larval stages and adults [96]. In cancer research, scRNA-seq has significantly advanced our understanding of the intrinsic mechanisms within intra- and inter-tumoural cells, and how these interactions influence tumour–host dynamics (Table 3).

Brain tumours. For neuroblast (NB) tumours induced by prosRNAi, Genovese et al. (2019) dissected ventral nerve cords (VNCs) from early adult female *D. melanogaster* [97]. Two heterogeneous populations were identified: Chinmo+Imp+ cells that could self-renew, propagate the tumour, evade quiescence, or reduce differentiation to Syp+E93+ cells. This transition involved the downregulation of glutamine and glucose metabolism genes, as confirmed by bulk RNA-seq and screening pathways, facilitating tumour progression. In another study on neuro stem cell (NSC)-derived brain tumours induced by *brat*^RNAi^, Bonnay et al. (2020) employed a multi-omics approach combining bulk RNA-seq, metabolomics, and scRNA-seq [36]. Bulk RNA-seq and metabolomics revealed increased glycolytic pathways and TCA intermediates, with glutamine as the primary energy source. Subsequently, scRNA-seq identified three cell clusters within the NSC tumours, which were categorised into proliferative and OxPhos-dependent cells, with high stress linked to unfolded protein response. These findings provided insights into the initiation and metabolic transition of tumourigenesis. Finally, using the same model but a different approach, Diaz et al. (2024) performed a single-cell-like analysis by extracting individual cells *in vivo* using capillaries, without enzymatic digestion, followed by differential expression analysis via microarrays. This study explored the transition from tumour-initiating cells (TIC) to brain cancer cells and discovered that increased OxPhos activity upregulates ribogenesis, mediated by the interaction between HEAT-Repeat Containing 1 (HEATR1) and Myc [98].

Wing disc model. In Ji et al.’s (2019) study, *scrib^1^* wing disc tumours exhibited a different growth rate compared to the wild type. Bulk RNA-seq analysis revealed an inverse relationship between decreasing OxPhos and increasing glycolysis from the early to late stages of tumour development. Furthermore, the JNK pathway was found to inhibit tumour growth, prompting the use of scRNA-seq to identify the specific cells responsible for this slowdown. The analysis revealed a heterogeneous population with varying expression levels distributed according to polarity, showing that tumour cells with high JNK levels were located near the surface of the tumour. Conversely, the main ERK pathway targets increased over time, promoting growth in the later stages. This indicates an antagonistic process where early-stage phenotypic growth arrest by JNK is eventually overridden by ERK overactivation, leading to the previously noted changes in the metabolic profile [99].

Eye disc model. Floc’hlay et al. (2023) utilised an injury-induced wing disc model to perform scRNA-seq, allowing for the identification of two distinct cellular clusters involved in pro-proliferative and pro-senescence pathways during tumourigenesis. After that, they compared these findings with *Ras^v12^ scrib^−/−^* eye tumour imaginal discs. The analysis revealed the presence of both proliferative and senescent cell populations within the eye tumour model, with a notable enrichment of pre-senescent cells. These pre-senescent cells were associated with pathways related to innate immunity, glutathione metabolism, cell migration, and irradiation response, suggesting that tumour cells in the eye model exhibit a persistent reactive injury response, which may contribute to the chronic nature of tumourigenesis in this context [100].

Salivary gland model. Khalili et al. (2023) adopted a different approach to studying tumour–host interactions by inducing *Ras^V12^*-driven tumourigenesis in the salivary gland. The focus of this study was on obtaining circulating and attached haemocytes for scRNA-seq, with the aim of characterising “healthy” tissues within the tumour context rather than the tumour cells themselves. Circulating haemocytes were collected by bleeding larvae and analysed using a modified version of the Smart-seq2 single-cell technique. The analysis resulted in the identification of tumour-associated haemocyte (TAH) profiles, which exhibited a significant immune activation signature. This finding suggests that TAHs may play a crucial role in mediating communication between tumour cells, haemocytes, and non-tumour tissues, potentially through exosomes carrying proteins, lipids, specific metabolites, and RNA [101].

In summary, different tissue tumour models are employed to investigate the various metabolic hallmarks of cancer. The majority of these studies focus on how tumour cells alter their functions to adapt to their environment and new molecular components. In contrast, there are studies, such as that by Khalili et al. (2023), where the primary focus is not on tumour cells themselves but rather on other “healthy” tissues or systems, such as the immune system [101]. Combining bulk RNA-seq and scRNA-seq is an effective strategy for identifying altered pathways and subsequently pinpointing the specific cell clusters responsible for these functions [36,99]. Furthermore, Bonnay et al. (2020) carried out a multidisciplinary approach integrating various omics techniques—such as proteomics and metabolomics—with bulk RNA-seq, scRNA-seq, and advanced high-resolution microscopy techniques like transmission electron microscopy (TEM). This study revealed how brain tumour cells undergo a transition that establishes a robust hierarchy defined by their metabolic profile, driven by crucial cellular processes like mitochondrial fusion [36].

**Table 3:** Summary of scRNA-seq in *D. melanogaster* cancer metabolism field.

D. melanogaster Tumour Model (Genotype)	Isolation of Single-Cell Process	Library/ Company	Reference
Salivary gland (*Ras^V12^*)	Non-tumour tissue: haemolymph bleeding larvae and capillary suction	Smart-seq2/Illumina (San Diego, CA, USA)	[101]
Eye disc (*Ras^V12^ scrib^−/−^*)	Eye tumoural disc digested by Dispase + Collagense mix	10XChromium (Pleasanton, CA, USA)	[100]
Wing disc (*scrib^1^*)	Wing tumour disc digested by trypsin-EDTA	10XGenomics (Pleasanton, CA, USA)	[99]
Brain (*pros^RNAi^*)	Ventral nerd cords (VNCs) were disaggregated by Collagenase + Papain mix	10XGenomics (Pleasanton, CA, USA)	[97]
(*brat^RNAi^*)	Central brains (CBs) without VNC were dissociated with the Collagenase + Papain mix	Smart-Seq2/Illumina (San Diego, CA, USA)	[36]
(*brat^RNAi^*)	Single cells extracted using capillarity *in vivo*	Qarray2/Genetix (New Milton, UK)	[98]

### 6.4. Dual Expression System in the Metabolic Cancer Context

Genetic tools in *D. melanogaster* have been widely used since the introduction of the GAL4/UAS system by Brand and Perrimon in 1993 [102]. Over the years, new systems such as LexA/LexAop and QF/QUAS have been developed, and the increasing complexity of the field has necessitated the combination of multiple systems to investigate various mechanisms underlying intercellular processes and inter-organ communication [103]. Among the numerous studies employing binary expression systems in neuroscience, developmental biology, and other areas, this review focuses on those utilising dual expression systems to explore metabolic changes in a tumour context. This approach is particularly useful for characterising systemic metabolic alterations in cancer, such as cachexia. These tools enable the interrogation of genes in key affected tissues, including muscle and the fat body.

Eye disc (QF/QUAS) + Muscle (GAL4/UAS). This research highlights the correlation between more neoplastic and invasive tumours and increased wasting and fat accumulation. Bulk RNA-seq in *Ras^V12^scrib^RNAi^* or *scrib*^1^ mutant larvae identified key factors driving cachexia, including the deregulation of stress, starvation, metabolic and inflammation responses, and immune regulatory genes within the cachectic muscle of tumour-bearing larvae. To elucidate the pathways involved in this metabolic degradation process, tissue-specific candidate genes were interrogated using a dual expression system. The QF/QUAS system was used to induce neoplastic invasive clones in the eye-antenna disc (*ET40*-QF > *QUAS-Ras^V12^ scrib^1^*), while the GAL4/UAS system was employed to assess muscle-specific gene expression under the *Mhc* driver. Inhibition of the JNK and JAK/STAT pathways provided minimal rescue from muscle wasting, suggesting their limited role in preventing muscle degradation [104]. Another study by Dark et al. (2024) use the eye disc tumour model (*ey-FLP*, *actQF2 Q > Ras^V12^scrib^RNAi^*) as developed by Lodge et al. (2021) [61]. This study focused more on the Mhc/Mef2-Gal4 system to target muscle genes, aiming to further investigate the tumour–muscle metabolic interaction. The research centred on mitochondrial function in muscle tissue, exploring pathways related to ROS, mitochondrial fission/fusion, Foxo signalling, autophagy, translation, lipid accumulation, and β-oxidation. The study demonstrated the significant role of mitochondrial fusion in muscle wasting and revealed that Foxo signalling is crucial for maintaining muscle integrity by regulating mitochondrial membrane potential and lipid metabolism via β-oxidation [57].

Eye disc (QF/QUAS) + Fat body (GAL4/UAS). Chen’s group induced eye-antennal disc tumours by recombining *ey-FLP*, *actGal4 Ras^V12^dlg^RNAi^*, resulting in cachexia symptoms. Firstly, Lodge et al. (2021) RNA-seq, combined with haemolymph proteomics and RNAi screening, identified various mediators responsible for disrupting the BM of the fat body. Among these, Mmp1 was found to activate TGF-β via Gbb in both the tumour and the fat body, leading to organ wasting. To precisely investigate the connection between the tumour and the fat body, a dual binary system was developed. In this system, eye disc tumours were induced using the QF/QUAS system (*ey-FLP*, *actQF2 Q > Ras^V12^ scrib^RNAi^*), while the GAL4/UAS system under the r4 driver was employed to screen genes of interest. The inhibition of TGF-β and Mmp1 in the fat body partially rescued organ wasting, indicating that TGF-β plays a central role in muscle detachment [61]. Bakopoulos et al. (2023), using the same dual system model, added that the secretion of TGF-β antagonist, short gastrulation (sog), and insulin signalling controls extracellular matrix (ECM) secretion. The interconnection between the fat body and muscle was further elucidated by demonstrating that disruption of the fat body’s BM contributes to muscle wasting [62].

## 7. Nutritional and Pharmacological Interventions in Metabolic Cancer

Metabolic adaptation in cancer cells is essential for progression and development. The features previously discussed across the three levels of cancer organisation contribute to tumour adaptation within a new environment, where intra- and extracellular interactions confer advantages to the tumour at the expense of the surrounding tissues. As tumours alter their metabolism to sustain overgrowth, new therapeutic strategies emerge to counteract these changes. This section will focus on how nutrition can modulate tumour progression and how, in some cases, the same dietary strategies or nutrients can exert opposite effects on the tumour and the host. Additionally, understanding the altered pathways exhibited by the tumour and developing specific metabolic inhibitors to halt its progression represents a promising area of research. The combination of using whole organisms, such as *D. melanogaster*, alongside pharmacological and nutritional treatments, provides a crucial foundation in the drug development process (Table 4).

### 7.1. Nutritional Interventions

The metabolic tumour changes discussed in the previous sections were developed using standard *D. melanogaster* diets. These diets typically contain corn flour, yeast, corn syrup, and agar. A prominent example is the Bloomington *Drosophila* Stock Center (BDSC) recipe, which includes yellow cornmeal, soybean meal, yeast, and light corn syrup. This diet is rich in carbohydrates and protein and is formulated to maintain healthy fly populations [105]. Alternative approaches include holidic diets, which are completely chemically defined and used for precise studies on nutrition and metabolism. These diets allow for the exact control of all nutritional components, eliminating the variability associated with natural ingredients. Defining and standardising food composition is critical to understanding the role that different nutrients play in tumourigenesis [106].

One of the most extensively studied diets is caloric restriction (CR), which is associated with various health benefits, including improved health outcomes, extended lifespan, and reduced morbidity and mortality rates in animal studies [107]. CR has also shown potential in cancer prevention. As a therapeutic approach for cancer, CR has been demonstrated to reduce tumour size and progression in many contexts by limiting the availability of nutrients that tumours can uptake [108,109]. Moreover, caloric restriction strategies have evolved to offer new benefits in cancer treatment. While chronic CR poses challenges and mixed effects, newer approaches like periodic fasting (PF), fasting-mimicking diets (FMDs), and dietary restriction (DR) without reducing calories show promise for cancer prevention and therapy [110]. In *D. melanogaster*, short cycles of DR, achieved by reducing the protein content (yeast, in the case of flies) relative to standard food, combined with full feeding periods, have been shown to reduce *Raf^gof^* gut tumour size, increase functionality, and extend lifespan [111]. In contrast, tumours with *Pten* loss exhibit resistance to DR, displaying aggressive and robust responses in both mice and flies [112,113]. The underlying mechanisms for this differential response remain unclear.

Focusing on specific nutrients such as carbohydrates, the impact of an HSD is noteworthy. As such, HSD can drive the transformation of benign *ras1^G12V^csk^−/−^* tumours into aggressive, invasive, metastatic, and lethal forms. This transition results in muscle wasting through the bnl-btl interaction, which releases amino acids, primarily proline. On the other hand, HSD upregulates the amino acid transporter *pathetic* via the Hippo pathway, creating a positive feedback loop that promotes tumour growth [69]. Additionally, HSD enhances tumour growth in *Hipk^OE^* tumours by allowing excess sugar to couple with the HIPK protein through OGT, further promoting tumour progression [26].

When analysing tumour behaviour in response to amino acids, manipulating methionine levels in wing disc tumours or histidine levels in brain tumours can either reduce or increase tumour size, respectively, through the previously discussed pathways in Section 2.3 [41,42]. As noted, tumours do not uniformly respond to the same interventions. For instance, brain tumours exhibit varying sensitivity to histidine levels; specifically, *nerfin-1^159^* and *N^OE^* are more responsive to histidine fluctuations, whereas *pros^RNAi^* shows less sensitivity [41].

In studies involving different diets or nutrient supplementation, a high-fat diet (HFD) using coconut oil or nicotinamide (Vitamin B3) supplementation in tumour-bearing animals (*QRas^V12^scrib^RNAi^* or *Ras^V12^dlg^RNAi^*) has been shown to partially reverse symptoms associated with cachexia. These dietary interventions help restore mitochondrial size, muscle integrity, and insulin signalling; enhance mitochondrial membrane potential; and improve cellular energy production, although they do not affect tumour size. Additionally, the regulation of β-oxidation of fatty acids, influenced by HFD, impacted in Withered (*Whd*), which is responsible for the increased utilisation of muscle lipids [57]. Notably, HFD interventions in mice can induce metabolic pathways promoting tumorigenesis, including breast cancer models, where HFD-induced gut microbiota activates the mTORC1 pathway, increasing immune-suppressive cells [114]. Additionally, in liver cancer, HFD enhances AKT activity via palmitoylation, driving hepatocellular carcinoma (HCC) [115]. Moreover, in intestinal cancer models, HFD has been shown to boost stem cell proliferation through PPAR-mediated fatty acid oxidation [116]. These observations highlight that HFD can have the opposite impact on cancers in different organisms, or, more likely, in different cancers driven by different oncogenes or in different organs or developmental stages, warranting future studies to expand the impacts of HFD on tumorigenesis using additional oncogene combinations, contexts, and life stages.

### 7.2. Pharmacological Treatments

Pharmacological treatments, including chemotherapy, immunotherapy, targeted therapy, hormone therapy, and metabolic inhibitors, have been extensively utilised in cancer treatment for many decades. The recognition of metabolic rewiring as a hallmark of cancer has intensified the focus on metabolic inhibitors [117]. In light of the impact of nutrition on cancer development, this review will highlight recent advancements in metabolic inhibitors within the *D. melanogaster* cancer research field. To assess the efficacy of these inhibitors, experiments were conducted using standard *D. melanogaster* food to correlate the drug’s effects with observed phenotypes. The following sections will discuss drugs targeting specific points in the rewire pathways, generally resulting in reduced tumour growth.

For instance, in the context of aerobic glycolysis, hydrazine (Hyd) inhibits phosphoenolpyruvate carboxykinase (PEPCK), which regulates glucose metabolism in *brat^RNAi^* brain tumour explants [118]. Excess glucose consumption often leads to pH acidification due to fermentation, which can be buffered by dipeptides such as L-carnosine (L-Car) and glycylglycine (GG), reducing folding phenotypes in *COX7a^RNAi^*, *Dl^OE^*, or *Ras^V12^* tumours [27].

Regarding mitochondrial targets, several inhibitors have been employed to revert tumour phenotypes and reduce tumour size. The RET process, previously discussed, can be biologically inhibited by adding NAM, CPT, and C-I30 ORF in *N^OE^* brain tumours [34]. Additionally, glycerol-3-phosphate uptake by mitochondrial glycerophosphate oxidase (GPO1) can be blocked by a specific inhibitor (iGP1), which reduces *brat^RNAi^* tumour explants [118]. Antioxidant treatments, such as N-acetyl cysteine (NAC), can partially restore the wild-type phenotype in *l(3)mbt^RNAi^* wing disc tumours by reducing ROS levels [119]. However, in *N^RNAi^* *mys^RNAi^* gut tumours, the overexpression of catalase to buffer ROS does not rescue tumour survival, suggesting that antioxidant treatments can sometimes exacerbate rather than alleviate tumour progression [39].

In terms of nucleotide metabolism, Ctps requires glutamine and UTP to generate CTP for pyrimidine production. The addition of the uridine analogue, 3-deoxyuridine, inhibits this enzyme and reduces tumour growth in *Ras^V12^* tumours [40]. Not all amino acid-targeted treatments are effective; for example, cimetidine, a histamine receptor inhibitor, does not affect tumour clone size in *nerfin-1^159^* [42]. Conversely, inhibitors of amino acid transporters, such as 2-amino-2-norbornanecarboxylic acid (BCH) and KYT0353 (JPH203), have shown efficacy in reducing tumour size by pharmacologically inhibiting of Jh-21 in *Ras^V12^scrib^−/−^* malignant tumours through mTOR signalling downregulation [43]. Furthermore, an HSD environment enhances the amino acid transporter Pathetic in *ras1^G12V^csk^−/−^* animals, while supplementation with Indole-3-propionic acid (IPA) dramatically suppresses tumour growth in a dose-dependent manner [69].

**Table 4:** Use of diet intervention or pharmacological inhibitors as treatments in *D. melanogaster* cancer metabolism.

Metabolic Modulator	Target	Effect	*D. melanogaster* Tumour Model	Reference
DR	Unknown	Reduce gut tumour size, increase functionality and lifespan	*Raf^gof^*	[111]
DR	Unknown	Resistant, aggressive, and tumour-like	*PTEN^117^*	[113]
HSD	Unknown	Mediate benign to neoplastic/metastatic transition Upregulating free amino acids and *pathetic* in the tumour	*ras1^G12V^csk^−/−^*	[69]
HSD	HIPCK	OGT couple sugar to HIPCK tumour growth	*Hipck^OE^*	[26]
Methionine	SAM	Incorporation of SAM to activate mTOR to induce cell proliferation	*Src42ACE*, *JNK DN*	[42]
Histidine	Hdc	Hdc converts histidine to histamine to Myc to improve ribogenesis, promoting tumour development	*nerfin-1^159^* and *N^OE^*	[41]
HFD	Unknown	Recovering mitochondrial size and membrane potential, muscle integrity, and insulin signalling	*QRas^V12^ scrib^RNAi^* or *Ras^V12^ dlg^RNAi^*	[57]
Nicotinamide (Vitamin B3)	Unknown	As HFD, but without changes in *whd* levels that regulate the β-oxidation of fatty acids	*QRas^V12^ scrib^RNAi^* or *Ras^V12^ dlg^RNAi^*	[57]
Hydrazine	PEPCK	Blocks PEPCK inhibiting glycolysis	*brat^RNAi^*	[118]
iGP1	GPO1	Glycerol-3-phosphate by mitochondrial GPO1 is blocked	*brat^RNAi^*	[118]
NAM, CPT and C-I30 ORF	RET	Revert tumoural phenotype and reduce size	*N^oe^*	[34]
L-Car and GG	pH	Buffer pH and reduce folding tumoural phenotype	*COX7a^RNAi^ Dl^OE^/Ras^V12^*	[27]
NAC	ROS	Reduce ROS from tumours with more folds	*l(3)mbt^RNAi^*	[119]
3-deoxyuridine	Ctps	Blocks pyrimidine production	*Ras^V12^*	[40]
Cimetidine	Histamine receptor	Blocks histamine receptor without changing tumour clone size	*nerfin-1^159^*	[42]
BCH and JPH203	Jh-21	Blocks Jh-21 reducing mTOR signalling	*Ras^V1 2^scrib^−/−^*	[43]
IPA	Pathetic	Suppressed tumour growth in a dose-dependent in HSD environment	*ras1^G12V^csk^−/−^*	[69]

## 8. Future Perspectives

Tumour metabolic adaptations are recognised as one of the most critical hallmarks of cancer evolution. We can better understand the complex oncogenic interactions by using various model organisms to explore the initiation of tumours *in situ*. In this context, *D. melanogaster* has emerged as a fundamental model in cancer initiation and progression research. As highlighted in this review, metabolic adaptations observed in humans are conserved in mice and also observed in fruit fly cancer. However, these changes still need to be more thoroughly explored; in particular, the role of lipid and nucleic acid metabolism in *D. melanogaster* tumour cells remains under-examined.

Regarding other external adaptations, we cannot understate the importance of investigating systemic metabolic processes related to organ wasting (cachexia) or accelerated ageing and frailty. Understanding the systemic symptoms of cancer is a crucial area of research that could significantly impact the survival and well-being of cancer patients, underscoring the urgency and significance of these studies. Additionally, we propose that tumour metabolism may influence the immune system beyond the well-studied ROS mechanisms, potentially opening new avenues for research.

Future studies using *D. melanogaster* models should also explore how metabolic reprogramming influences or facilitates metastatic potential. Emerging evidence suggests that an altered metabolism may support tumour initiation, growth, and the invasion of surrounding tissues and formation of distant metastases.

The advent of new technologies—such as metabolic sensors, dual expression systems, and advanced omics—has opened up exciting possibilities for cancer initiation research. These tools have allowed us to delve deeper into how metabolites and metabolic pathways adapt to the demands of tumours, sparking inspiration for further exploration. Furthermore, emerging nutritional and pharmacological interventions present promising opportunities for future therapies. Specific diets and drug treatments tailored to particular oncogenic-driven tumours and contexts could slow disease progression or enhance cancer therapy responses.

## 9. Conclusions

The fruit fly *D. melanogaster*, a seemingly simple organism, holds immense potential as a model for studying cancer metabolism. It allows us to delve into the intricate levels of cancer, from internal metabolic adaptations to the systemic effects on non-cancer tissues and organs and even the metastatic process. The fact that many of the changes associated with cancer in zebrafish, mice, and humans are also present in the fruit fly is a testament to the power of this model. It highlights that the cancer process is not a modern affliction but an ancient disease, as evidenced by its presence in some ancient human bones.

Cancer, a relentless energy-demanding process, necessitates a constant supply of energy and metabolites to fuel continuous cell proliferation and the construction of cell membranes. This very metabolic demand of cancer cells has been proposed as a potential Achilles’ heel in our battle against cancer progression, offering a ray of hope in our fight.

Metabolic interventions, whether through diet or pharmacological means, have emerged as good strategies in the fight against tumorigenesis, alleviating its side effects or even potentiating the effects of other interventions or medicines. However, recent studies also emphasise that the effectiveness of these strategies highly depends on the specific oncogene driver, the context, and even the host’s genotype. These observations underscore the importance of future personalised medicine in which the host is taken into account to predict therapy response and outcome. Understanding how the same metabolic intervention, such as the HFD, can yield opposite results in different scenarios or in different internal states of the host will provide insights to more effectively manipulate the metabolism to achieve a positive effect on the host and reduce the cancer cells.

Key underexplored pathways—such as lipid metabolism, fatty acid oxidation, and the pentose phosphate pathway—are promising targets for identifying drugs or dietary changes that can influence tumorigenesis and enhance cancer treatment strategies. Screenings in fruit flies can streamline the repurposing of FDA-approved drugs, providing a valuable basis for similar studies in less genetically amenable organisms. 

## Figures and Tables

**Table 1:** Metabolic sensors to study internal metabolic changes in tumour cells.

Metabolic Sensor	Molecule Target	Technology	Reference
Glucose sensor	Glucose	GAL4/UAS system coupled with FRET	[25]
Laconic	Lactic acid	GAL4/UAS system coupled with FRET	[28]
Pyronic	Pyruvate	GAL4/UAS system coupled with FRET	[28]
OGsor	2-oxoglutarate	GAL4/UAS system coupled with FRET	[28]
Sonar	NADH/NAD	Dual excitation/single emission sensors	[31]
PercvialHR	ATP/ADP	Dual excitation/single emission sensors	[31]
gstD-GFP	H_2_O_2_	Mono-fluorescent sensors coupled to GFP	[23]
MitoRoGFP2_Orp1	H_2_O_2_	Mono-fluorescent sensors coupled to GFP	[31]
MitoRoGFP2_Grx1	Glutathione	Mono-fluorescent sensors coupled to GFP	[31]
pH sensor	pH	Mono-fluorescent sensors coupled to GFP	[27]
2-NBDG	Mimics glucose	Tracking glucose uptake by a fluorescent analogue	[25]

## Data Availability

Data sharing is not applicable to this article.
